# Identification of Proteins Associated with Multilamellar Bodies Produced by *Dictyostelium discoideum*

**DOI:** 10.1371/journal.pone.0158270

**Published:** 2016-06-24

**Authors:** Alix M. Denoncourt, Valérie E. Paquet, Ahmadreza Sedighi, Steve J. Charette

**Affiliations:** 1 Institut de Biologie Intégrative et des Systèmes, Pavillon Charles-Eugène-Marchand, Université Laval, Quebec City, QC, Canada; 2 Centre de recherche de l’Institut universitaire de cardiologie et de pneumologie de Québec, Hôpital Laval, Quebec City, QC, Canada; 3 Département de biochimie, de microbiologie et de bio-informatique, Faculté des sciences et de génie, Université Laval, Quebec City, QC, Canada; MRC Laboratory of Molecular Biology, UNITED KINGDOM

## Abstract

*Dictyostelium discoideum* amoebae produce and secrete multilamellar bodies (MLBs) when fed digestible bacteria. The aim of the present study was to elucidate the proteic content of MLBs. The lipid composition of MLBs is mainly amoebal in origin, suggesting that MLB formation is a protozoa-driven process that could play a significant role in amoebal physiology. We identified four major proteins on purified MLBs using mass spectrometry in order to better understand the molecular mechanisms governing MLB formation and, eventually, to elucidate the true function of MLBs. These proteins were SctA, PhoPQ, PonC and a protein containing a cytidine/deoxycytidylate deaminase (CDD) zinc-binding region. SctA is a component of pycnosomes, which are membranous materials that are continuously secreted by amoebae. The presence of SctA on MLBs was confirmed by immunofluorescence and Western blotting using a specific anti-SctA antibody. The CDD protein may be one of the proteins recognized by the H36 antibody, which was used as a MLB marker in a previous study. The function of the CDD protein is unknown. Immunofluorescence and flow cytometric analyses confirmed that the H36 antibody is a better marker of MLBs than the anti-SctA antibody. This study is an additional step to elucidate the potential role of MLBs and revealed that only a small set of proteins appeared to be present on MLBs.

## Introduction

Multilamellar bodies (MLBs) are structures of lysosomal origin composed of multiple concentric membrane layers [1]. They are produced by various types of eukaryotic cells, including protozoa such as *Dictyostelium discoideum*, which are soil organisms that feed mainly on bacteria by phagocytosis. They produce MLBs ranging in size from 0.5 to 2 μm and secrete them into the environment [2–4].

*D*. *discoideum* MLBs are produced in abundance when the cells are grown in the presence of digestible bacteria but are virtually absent when the cells are grown in nutrient liquid medium [3, 5–7]. Contrary to what was suggested in the literature, MLBs do not appear to be a waste disposal system that serves only to eliminate undigested bacterial remains. They are likely formed by repetitive inward budding of the membrane of lysosomal compartments [6, 7]. Moreover, based on biochemical analyses of purified MLBs, it appears that lipids in MLBs are mainly amoebal in origin rather than being similar to the bacterial lipid profile. These results indicate that MLB membranes are the product of amoebal metabolism [3]. Hence, even if digestible bacteria are required for *D*. *discoideum* to produce MLBs, the process depends largely on the metabolic capability of the amoebae. MLBs may thus play a significant albeit unknown role in amoebal physiology.

MLBs, which are also called expelled vesicles and fecal pellets, are produced by various types of protozoa and are also involved in the bacteria packaging process, a phenomenon observed when ingested bacteria can resist enzymatic degradation that normally occurs in the phago-endocytic pathway before being packaged in MLBs or related structures. To date, viable packaged bacteria have been observed in the case of five bacterial pathogenic species, including the respiratory pathogen *Legionella pneumophila* (reviewed in [7]. Bacteria packaged in vesicles are more resistant to a variety of stresses, including biocides and antibiotics [8–11]. The bacteria packaging process may thus be involved in the persistence and transmission of some pathogenic bacteria. It has been suggested that bacteria-containing MLBs would also be small enough to be aerosolized and to be inhaled by humans [8].

Given that MLB formation is under the control of the protozoa, the elucidation of the molecular mechanisms governing this process would provide a better understanding of the bacteria packaging phenomenon. This objective cannot be achieved without a more extended knowledge of the biochemical composition of MLBs and, more specifically of the protozoal proteins associated with these structures. Identifying these proteins and their functions may provide clues to the physiological roles of MLBs. Some proteins have already been identified on *D*. *discoideum* MLBs, including discoidin I and a cysteine proteinase, as well as yet unidentified glycosylated proteins [12–14]. However, discoidin I appears to be associated with MLBs solely in specific circumstances related to multicellular development [12, 13].

In the present study, we used a proteomic approach to identify four major proteins on purified MLBs, including SctA and a protein containing a cytidine/deoxycytidylate deaminase (CDD) zinc-binding region. Based on immunoprecipitation and mass spectrometric analyses, the CDD protein may be one of the epitopes recognized by the H36 antibody [15]. This antibody has been used as an MLB marker [3], but its epitope is unknown.

## Materials and Methods

### Amoeba, bacteria, and antibodies

*D*. *discoideum* DH1-10 cells [16] were grown at 21°C in HL5 medium supplemented with 15 μg/mL of tetracycline as previously described [15]. They were subcultured twice a week in fresh medium to prevent the cultures from reaching confluence. They were also grown on bacterial lawns as described below.

*K*. *aerogenes* bacteria were grown on LB agar (Millipore, USA) at 37°C, typically for 24h, before being used for the bacteria/amoebae co-culture experiments.

The H36 antibody (monoclonal mouse antibody) has been previously described [15]. The anti-SctA antibody (B4.2) is a monoclonal mouse antibody [17]. Both antibodies were kindly provided by P. Cosson. The AD7.5 mouse monoclonal antibody, which recognizes N-acetylglucosamine-1-phosphate, was a gift from Hudson H. Freeze (Sanford-Burnham Medical Research Institute, La Jolla, CA, USA) [18].

### Bacteria/amoebae co-cultures and purification of MLBs

2 x 10^6^ *D*. *discoideum* cells were grown with *K*. *aerogenes* on HL5 agar in 15 cm Petri dishes for 7 days at 21°C to obtain large phagocytic plaques. Samples of the phagocytic plaques collected using sterile pipette tips were diluted in fresh HL5 medium and were processed for immunofluorescence as described below.

*D*. *discoideum* cells were grown in the presence of *K*. *aerogenes* to induce them to secrete large amount of MLBs. The amoebae and bacteria were mixed in a proportion of 1:1000, respectively. They were plated on HL5 agar and were incubated at 21°C. After 5 or 6 days, the bacterial lawn was almost entirely consumed by the *D*. *discoideum* cells. At this point, the co-culture was harvested using a sterile scraper, resuspended in 10 mL of starvation buffer (2 mM Na_2_HPO_4_, 14.7 mM KH_2_PO_4_, 100 mM sorbitol, 100 μM CaCl_2_, and 1% HL5) [19], and centrifuged at 450 x g for 5 min to pellet the amoebae. The supernatant was mixed with 5 x 10^7^ *D*. *discoideum* cells freshly grown in liquid HL5. The new co-culture was shaken at 200 rpm overnight at 21°C and was centrifuged at 450 x g for 5 min. The supernatant contained MLBs and particles (<0.5 μm) of various appearances. To concentrate the MLBs and separate them from the particulate material, 1 mL of supernatant was deposited on a 6-mL sodium bromide gradient (pH 5) ranging in density from 1.0 to 1.5 g/mL in a glass tube. The tube was centrifuged at 3220 x g for 45 min at room temperature. The yellowish aggregate corresponding to the pure MLB fraction [3] was collected using a Pasteur pipette and was transferred to a 1.5-mL tube. The purified MLBs were washed twice with PBS by centrifuging them at 17,000 x g for 10 min between each wash. The protein concentration of the pelleted MLBs was determined using the Quick Start^™^ Bradford Protein Assay kit 1 (Bio-Rad, Canada). The purified MLBs were stored at 4°C in a small volume of fresh 1x PBS. The purified MLBs were examined by transmission electron microscopy (TEM) as previously described [3].

### Protein extraction from purified MLBs

The pellet of purified MLBs was resuspended in denaturation solution to obtain a concentration of 0.5 μg/μL. Two different denaturation solutions (DS1 and DS2) were used to determine whether the composition of the solution had an impact on the identification of the proteins. The DS1 solution was composed of 10 M urea, 2% CHAPS, 50 mM DTT, and 1 M thiourea. The DS2 solution was composed of 10 M urea, 2% CHAPS, and 200 mM DTT. The proteins were solubilized and denatured at 95°C for 5 min. The samples were mixed by inversion for 1 h and were centrifuged at 17,000 x g for 10 min. One-half volume of 3x TEX loading buffer (0.22 M Tris, pH 6,8, 23.5% glycerol, 9% SDS and traces of Bromophenol blue) was added to obtain a final concentration of 1x TEX. The lipidic structures were disrupted using a syringe and a 26G needle, and the mixture was heated at 95°C for a further 5 min. The heating steps were omitted for samples that were to be assessed by Western blotting with the anti-SctA and H36 antibodies.

### SDS-PAGE and Western blot analyses

The solubilized proteins were separated on 10% SDS-PAGE or 4–20% nUView Tris-Glycine gradient gels (NuSep, USA) in reducing (5% (v/v) 2-mercaptoethanol added to the samples loaded on the gel) or non-reducing conditions. Four major protein bands were excised from the gels run in non-reducing conditions for mass spectrometric analyses (see below). Samples were also separated on 12% SDS-PAGE gels for mass spectrometric analyses of total MLB proteins. Gels run in reducing conditions were used for Western blotting with the anti-SctA, H36, and AD7.5 antibodies. Gels run in non-reducing conditions were also used for Western blotting with H36 and AD7.5 antibodies.

Protein bands were electrotransferred to nitrocellulose membranes, which were immersed in 50 ml TBS (10 mM Tris, pH 7.4, and 150 mM NaCl) for 5 min. The membranes were incubated with TBSM (10 mM Tris, pH 7.4, 150 mM NaCl, and 7% skim milk) overnight at 4°C to block non-specific binding. The membranes were washed five times for 5 min with TBST (10 mM Tris, pH 7.4, 150 mM NaCl, and 0.1% Tween 20) and were incubated for 90 min at room temperature with the anti-SctA antibody (undiluted hybridoma supernatant), H36 (ascite diluted 1:20,000 in TBST), or AD7.5 antibody (hybridoma supernatant diluted 1:50 in TBST). They were washed three times for 5 min in TBST and were incubated for 1 h at room temperature with peroxidase-conjugated goat anti-mouse IgG (Sigma-Aldrich, Canada) or goat anti-mouse IgG IRDye 680RD (Li-cor, USA). They were washed six times for 5 min in TBST. The protein bands were revealed using an ECL chemiluminescent detection reagent (Millipore, USA), and images were acquired using photosensitive films or were directly acquired with the Odyssey Fc Imaging System (Li-cor, USA).

### Immunofluorescence

Samples containing axenic cells or cells and material from the periphery of phagocytic plaques resuspended in HL5 were allowed to adhere to glass coverslips for 60 min and were then fixed for 30 min in 4% paraformaldehyde. The coverslips were rinsed with PBS containing 40 mM NH_4_Cl and then with PBS. The samples were permeabilized for 5 min with PBS containing 0.1% saponin, and the coverslips were then immersed in PBS containing 0.2% bovine serum albumin (PBS-BSA) at room temperature for at least 5 min to block non-specific binding sites. The samples were incubated for 45 min with the appropriate primary mouse antibody (1:3 anti-SctA or 1:1000 H36 in PBS-BSA containing 1:200 Alexa 568-coupled phalloidin (Thermo Fisher, Canada)). The samples were rinsed with PBS-BSA and were incubated with an Alexa 488-coupled mouse IgG secondary antibody (Thermo Fisher, Canada). The coverslips were then mounted on glass slides using Prolong Gold (Thermo Fisher, Canada). Images were acquired using an Axio Observer Z1 microscope equipped with an Axiocam camera (Carl Zeiss, Canada).

For the flow cytometric analyses, purified MLBs were labeled with the primary anti-SctA or H36 antibody and then the secondary Alexa 488-coupled antibody as described above. The samples on the coverslips were suspended in PBS and were analyzed using a Guava easyCyte^™^ 8HT Sampling Flow Cytometer (Millipore, Canada).

### Immunoprecipitation using the H36 antibody

*D*. *discoideum* cells (approx. 2x10^7^) grown in liquid HL5 medium were centrifuged for 5 min at 1400 x g. They were resuspended in 5 mL of lysis buffer (PBS containing 1% NP-40, 1 mM EDTA, and 1 mM phenylmethylsulfonyl fluoride [PMSF]) and were kept on ice for 20 min. The cell lysate was centrifuged at 13,000 x g for 15 min at 4°C. Protein A sepharose beads or Protein G sepharose beads (200 μL; Sigma-Aldrich, Canada) were washed three times with 1 mL of lysis buffer without PMSF. The beads were resuspended in 300 μL of lysis buffer. A portion of the beads (100 μL) was stored at 4°C while 200 μL was mixed with 800 μL of lysis buffer and 50 μL of H36 antibody. The mixture was shaken for 1 h at 4°C and was then washed twice with lysis buffer. The H36 antibody-bound beads were resuspended in a final volume of 200 μL of lysis buffer and were mixed (50 μL of Protein A- or G sepharose beads) with 650 μL of cell lysate or 650 μL of lysis buffer (control). An aliquot (50 μL) of the beads not bound to H36 antibody was also mixed with 650 μL of cell lysate as a second control. The mixtures were prepared in duplicate, incubated for 24 h at 4°C, and washed three times with lysis buffer.

Immunoprecipitated proteins were separated by SDS-PAGE on a 10% acrylamide gel under non-reducing conditions without boiling the samples. The gel was stained with Coomassie blue to reveal the proteins. Multiple bands were visible on the gels, with a major band at 47 kDa, as previously observed [15]. The 25–35, 47, and 75-kDa protein bands were excised.

### Protein identification by mass spectrometry

The identity of the proteins excised from the SDS-PAGE gels (purified MLBs and H36 immunoprecipitation) was determined by matrix-assisted laser desorption/ionization time-of-flight mass spectrometry (MALDI-TOF-MS) at the CHUL proteomic platform (CHUL, Quebec City, QC, Canada). The MALDI-TOF-MS was performed twice following the immunoprecipitation procedure using Protein A sepharose beads and once following the immunoprecipitation procedure using Protein G sepharose beads to ensure the validity of the protein identification. In the case of purified MLBs, the complete set of solubilized proteins was analyzed three times with three different preparations of purified MLBs. Specific protein bands excised from the SDS-PAGE gels were analyzed once or twice depending on the first result.

## Results

### Identification of proteins associated with MLBs

The extraction and solubilization of the MLB-associated proteins require the use of solutions containing high concentrations of chaotropic agents such as urea because they were embedded in compact multilamellar lipidic layers. MLBs are mainly composed of amoebal lipids [3] and, as such, they could not be loaded on SDS-PAGE gels without undergoing a solubilization step with the appropriate buffer. Several chaotropic agents and detergents were used based on the denaturation solutions commonly used for protein electrophoresis. These agents break hydrogen and disulfide bonds and disrupt van der Waals forces and non-covalent interactions between proteins and non-proteinaceous compounds such as lipids [20]. For example, urea maintains proteins in their denatured state and keeps them soluble, while CHAPS promotes solubilization and minimizes protein aggregation [21]. Thiourea was also added because it was suspected that large amounts of protein would be solubilized and it was necessary to prevent them from precipitating, which would have resulted in poor resolution due to smeared bands [22]. The denaturation solution disrupted the 3-D conformation of the proteins and, more importantly, enabled us to separate the proteins from the lipids without disturbing the amino acid chains [20]. After solubilization, the MLB proteins were separated on SDS-PAGE gels and were excised for mass spectrometric analyses.

The list of MLB proteins identified by mass spectrometry is given in Table 1. This table does not include actin, tubulin, mitochondrial proteins, histones, peroxiredoxin, phosphoglycerate mutase, ribosomal proteins, and elongation factors, which were intentionally left off the list. These are the proteins that are most frequently detected in mass spectrometric analyses regardless of the sample type [23, 24] and would likely be false positives. The presence of actin was still assessed by fluorescent phalloidin staining of purified MLBs, with negative results (data not shown). The complete list of identified proteins is given in S1 Table. The remaining proteins were ranked based on the number of times they were identified in three mass spectrometric analyses of different preparations of purified MLBs. These proteins were detected with the two denaturation solutions, suggesting that the proteomics results were consistent regardless of the protein solubilization method used. Four proteins were found in the MLB preparations in each analysis. These proteins were, in decreasing proportion, PonC (ponticulin-like proteins) (Q54LC2, Q54LC3, Q54LC0, Q54LC1, Q54LB9), PhoPQ (Q86JB6), SctA (O77257), and the product of gene DDB_G0292096 (Q54DP5), which will be referred to as CDD. PonC proteins (PonC1 to 5) are almost identical 15-kDa actin-binding proteins associated with the plasma membrane [25]. Since all detected peptides were obtained in common regions of the PonC proteins, it was not possible to distinguish between the five. PhoPQ is a hypothetical protein similar to AprA, an autocrine repressor of proliferation [26], and SctA is an 18-kDa protein with an unknown function and a predicted peptide signal. The initial characterization of this protein is shown in a companion article [17]. The fourth protein, based on the information in dictybase.org, contains a cytidine and deoxycytidylate deaminase (CDD) zinc-binding region, a carbohydrate-binding domain, and a predicted peptide signal.

**Table 1:** Major proteins associated with MLBs based on mass spectrometric analyses.

Protein	Description	Gene ID	Molecular weight (kDa)
**PonC**	Ponticulin-like proteins	DDB_G0286717	15
		DDB_G0286715	
		DDB_G0286721	
		DDB_G0286719	
		DDB_G0286723	
**PhoPQ**	PhoPQ-activated pathogenicity-related protein	DDB_G0276897	56
**SctA**	Secreted Protein A	DDB_G0278725	18
**CDD**	Cytidine/deoxycytidylate deaminase zinc-binding domain-containing protein	DDB_G0292096	35

The table does not include ribosomal proteins, elongation factors, actin, tubulin, mitochondrial proteins, peroxiredoxin, phosphoglycerate mutase, or histones, which commonly generate false positives in proteomic studies [23, 24]. Only proteins identified in three independent analyses are shown.

**Fig 1:**
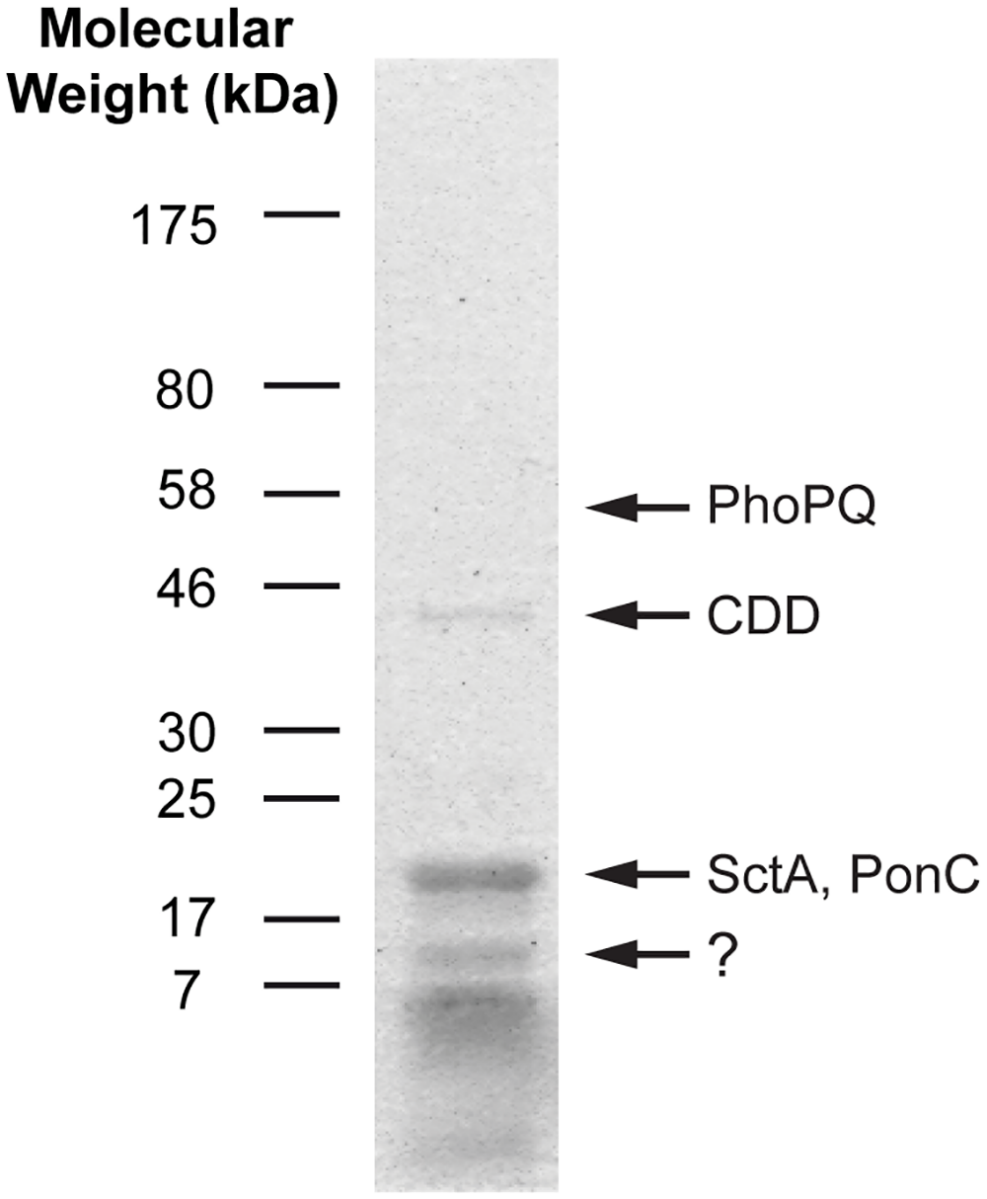
Protein profile of purified MLBs. Proteins were extracted from purified MLBs, solubilized with denaturation solutions, and run on an SDS-PAGE gel. The gel was stained with Coomassie blue to reveal the proteins. Most of the proteins had low molecular weights. Only a few major bands are visible on the gel. Based on mass spectrometric results, the 18-kDa band was a mix of the SctA and PonC proteins, while the 46 and 56-kDa bands corresponded to the CDD and PhoPQ proteins, respectively. The 10-kDa band gave inconclusive results.

The protein profile of purified MLBs is shown in Fig 1. The molecular weights of the most abundant proteins were less than 25 kDa. Four bands were excised from the gels for identification by mass spectrometry. The band at ~10 kDa could not be identified while the band at ~18 kDa was composed of SctA and PonC. The CDD protein was identified in the 46 kDa band. The slice at 56 kDa was also excised because it corresponded to the molecular weight of one of the major proteins detected in the total MLB extract. As expected, the PhoPQ protein was identified in the 56 kDa band. The molecular weights of proteins did not necessarily correspond to the molecular weights of the associated band. This may have been due to post-translational modifications of the proteins (such as glycosylation or cleavage), which would have affected their migration on the gel.

### Confirmation of the presence of SctA on MLBs

**Fig 2:**
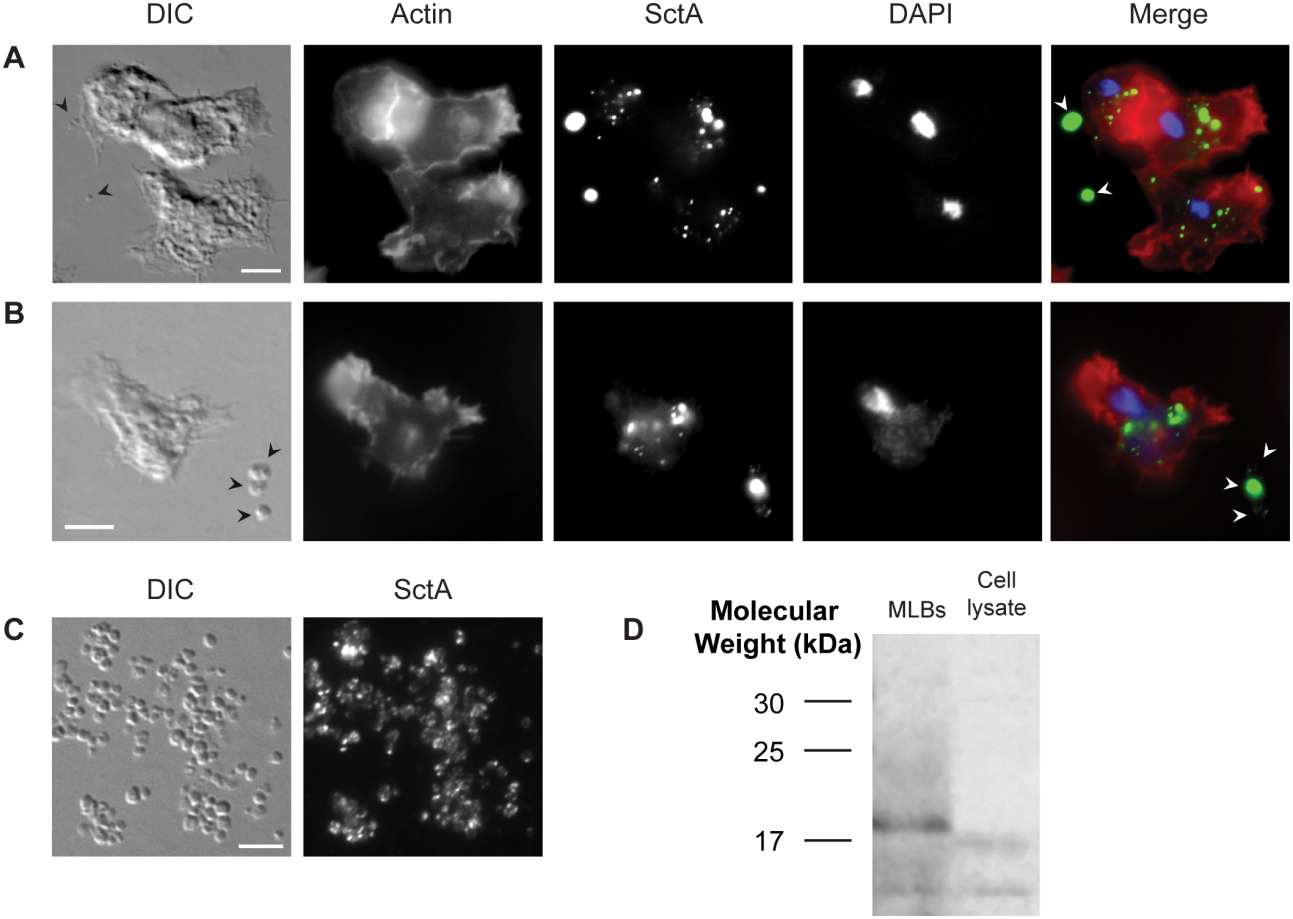
SctA is an MLB-associated protein. Immunofluorescence analyses using the anti-SctA antibody on (**A**) axenic cells, (**B**) cells grown with bacteria, and (**C**) purified MLBs. The cell cultures and purified material were deposited on glass slides. Nuclei or bacterial DNA were stained with DAPI, actin was stained with fluorescent phalloidin, and the cells were labeled using the anti-SctA antibody and a peroxidase-conjugated secondary antibody. In axenic conditions, SctA was observed in dot-like structures within the cells and on secreted pycnosomes. In the presence of bacteria (not visible on the photograph), SctA was found on extracellular MLBs, which are larger structures than pycnosomes. Some of the MLBs did not appear to be SctA-positive. Purified MLBs were all labeled with the anti-SctA antibody but the intensity of fluorescence was uneven. The arrows indicate MLBs and pycnosomes. (**D)** Western blot analysis with the anti-SctA antibody. The protein was detected in ~10 and 18-kDa bands in the MLB sample and in 10 and 17-kDa bands in the cell lysate (500,000 axenically grown cells). The purified MLB and cell lysate samples contained 5% 2-mercaptoethanol and denaturation solution. Scale bar: A to C = 5 μm.

Since SctA was identified in each of the mass spectrometric analyses and since a specific anti-SctA monoclonal antibody was available [17], the association of SctA with MLBs was verified by immunofluorescence and Western blot analyses. The cellular localization of the protein was also assessed in axenic conditions and in the presence of digestible bacteria (*K*. *aerogenes*), which are required for the production of MLBs. Fig 2A shows epifluorescence microscopic images of *D*. *discoideum* cells grown axenically that were stained with DAPI and phalloidin (actin), and labeled with anti-SctA antibody. Several SctA-positive dots were visible in the *D*. *discoideum* cells (as described by Sabra et al. [17]), and some of these dots appeared to be enclosed in actin-positive post-lysosomes. Post-lysosomes can be distinguished from lysosomes since actin accumulates on the former but not on the latter [27, 28]. Interestingly, SctA-positive structures were also observed in the extracellular medium despite the fact that *D*. *discoideum* does not produce MLBs in the absence of digestible bacteria. These structures are likely pycnosomes, which are membranous materials that are continuously formed in endosomal compartments and are secreted by the cells. SctA is known to be a marker of pycnosomes in *D*. *discoideum* cells [17]. The strong fluorescence of the extracellular dots suggested that these small structures contain large concentrations of SctA.

**Fig 3:**
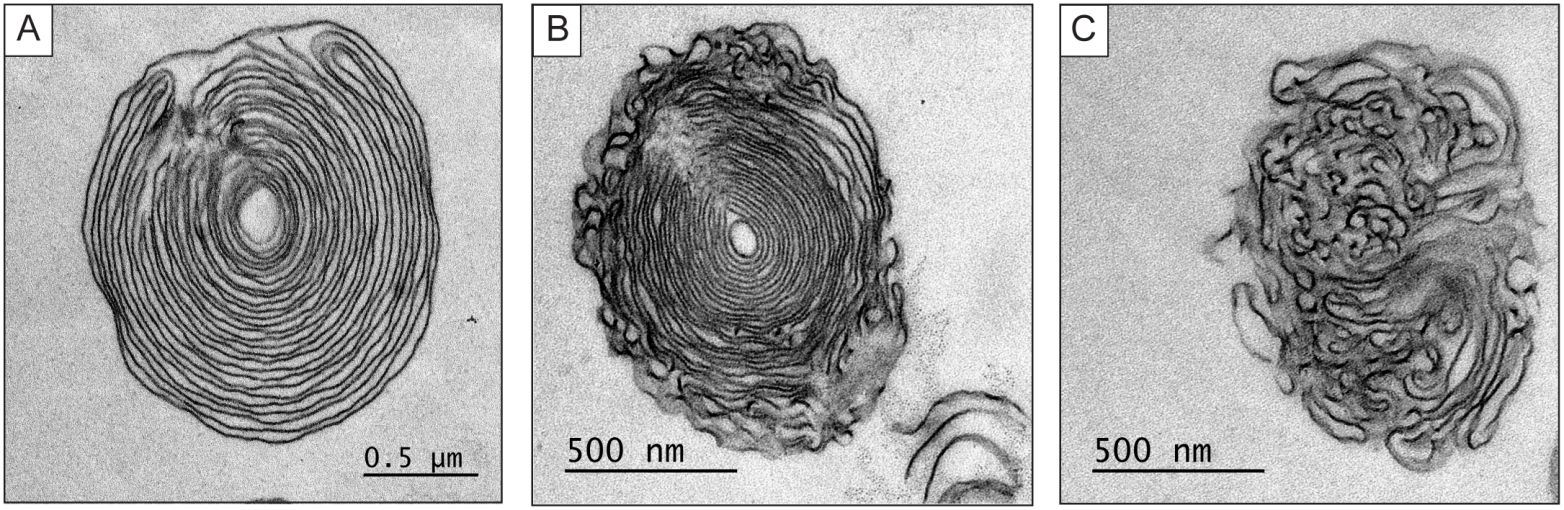
MLBs have different morphologies. Transmission electron microscopic images of MLBs composed of (**A**) concentric membrane layers, (**B**) a mix of concentric membrane layers and amorphous material, and (**C**) amorphous material. Scale bar: **A** to **C** = 0.5 μm.

In the presence of *K*. *aerogenes*, *D*. *discoideum* produced SctA-positive extracellular MLBs, which can be distinguished from pycnosomes by their larger size (Fig 2B). Several MLBs were detected by differential interference contrast microscopy (DIC) but not all of them were SctA-positive. SctA was also observed in dot-like structures inside the amoebae. Purified MLBs are shown in Fig 2C. While most of the structures appeared to be SctA positive, the intensity of the fluorescence was uneven. This might explain why some of the MLBs (produced in the presence of *K*. *aerogenes*) shown in Fig 2B were not SctA-positive. In addition, in samples containing amoebae, bacteria, and debris, it was harder to distinguish the weak signal of MLBs with fewer SctA proteins. The SctA labeling was also concentrated in dots rather than being spread out uniformly in the MLBs. It is possible that more or less compact pycnosomes are embedded in the MLBs, which would explain the faint labeling of MLBs by the anti-SctA antibody. Indeed, some MLBs exhibited regions of non-concentric membrane layers (Fig 3), which are similar to the pycnosome structures inside the cells (See Sabra et al. [17]). It has been shown that SctA-positive structures, likely pycnosomes, are incorporated into intraendosomal membranes in cells treated with U18666A [17], a drug that induces the formation of multilamellar structures in endosomes [6].

The presence of SctA on purified MLBs was also assessed by Western blotting. The anti-SctA antibody (Fig 2D) detected two bands (~10 and 18 kDa), one of which corresponded to the molecular weight of SctA (18 kDa). The mass spectrometric analysis confirmed that the 18-kDa band was indeed SctA. The Western blotting analysis also confirmed the presence of the two bands associated with SctA in the cell lysate, although the protein was detected in much lower quantity. This was not surprising given that, in axenic conditions, the protein is mostly found in the supernatant (possibly associated with pycnosomes) while much less is present in the cells [17]. One of the bands detected in the cell lysate was slightly lower than in the MLB sample. It is possible that post-translational modifications are added to the SctA protein when included in MLBs, which would change its apparent molecular weight.

### The H36 antibody recognizes many proteins, including one on MLBs

**Fig 4:**
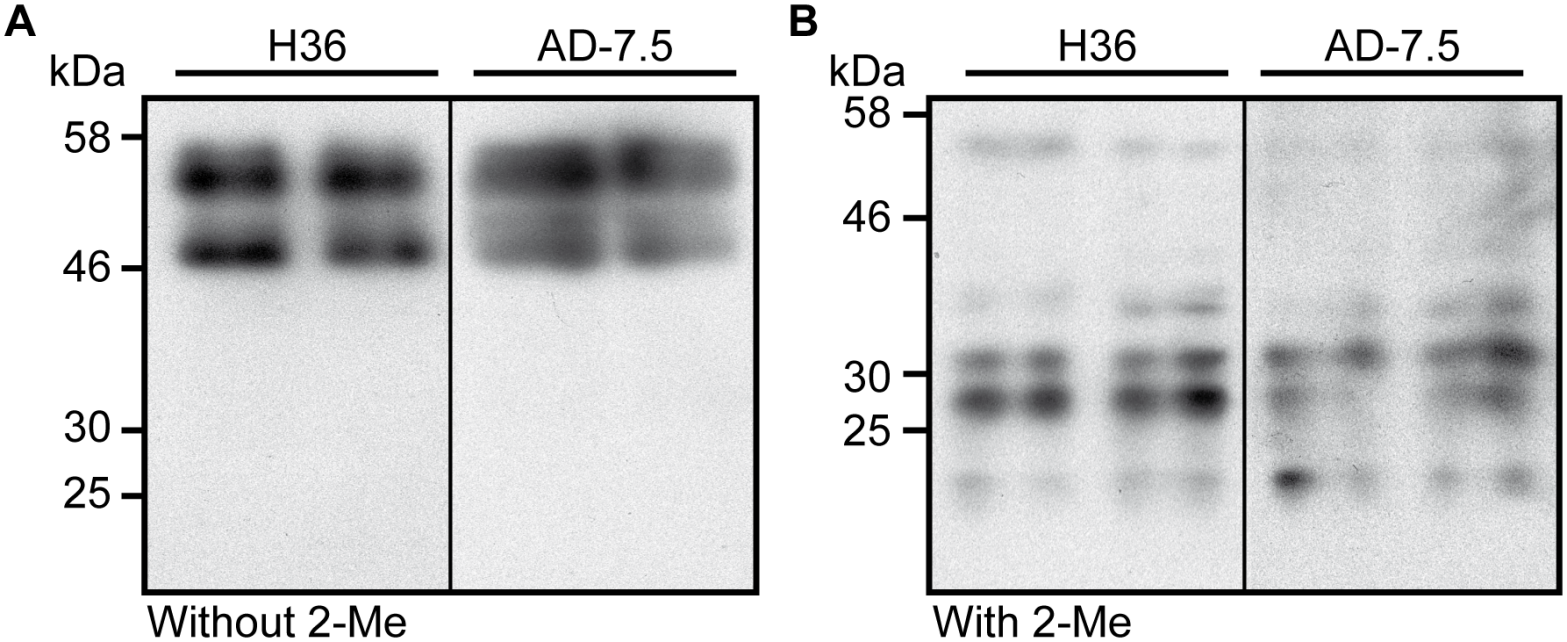
The H36 and AD7.5 antibodies recognize the same protein bands in reducing and non-reducing conditions. A total cell extract of *D*. *discoideum* cells grown in liquid HL5 medium was prepared in the (A) absence or (B) presence of 2-mercaptoethanol (2-Me). The extract was separated by SDS-PAGE, transferred to nitrocellulose membranes, and two tracks of each membrane were blotted with the H36 or AD7.5 antibody. This experiment was repeated twice with identical results.

An immunoprecipitation approach performed on total cell lysates combined with a mass spectrometry analysis was used to identify the nature of the antigen recognized by the H36 antibody. The main protein detected by this analysis was cysteine proteinase 7 (CP7), or proteinase-1, which is a 47-kDa protein encoded by the *cprG* gene [29]. However, CP7 was never detected by the mass spectrometric analyses performed on purified MLBs (Table 1). CP7 is the principal proteolytic enzyme in *D*. *discoideum* vegetative cells [18, 30] and is also the main protein recognized by the AD7.5 monoclonal antibody, which binds to N-acetylglucosamine-1-phosphate [18]. Since the H36 antibody also recognizes many proteins, a Western blot analysis of a total extract of *D*. *discoideum* cells was performed in parallel using both antibodies. Interestingly, the migration profiles of the proteins detected by the AD7.5 antibody were different in reducing and non-reducing conditions [31]. In non-reducing condition, the antibody recognized two major bands (47 and 55 kDa) while, in reducing conditions, multiple smaller molecular weight bands were detected (Fig 4), as previously described [29, 31], suggesting that the two bands detected in non-reducing conditions may contain many different proteins. As shown in Fig 4, the H36 and AD7.5 antibodies both generated the same band profiles in reducing and non-reducing conditions, confirming that the antigen recognized by the H36 antibody is also N-acetylglucosamine-1-phosphate, a post-translational modification that can be found on many proteins. These results also suggested that a protein other than CP7 was detected on the MLBs by the H36 antibody, which would explain why CP7 was not identified on MLBs by mass spectrometry. Interestingly, the CDD protein, which was one of the four major proteins associated with purified MLBs, was also detected by immunoprecipitation with the H36 antibody.

A Western blot was performed on purified MLBs using the H36 antibody to determine whether the CDD protein is the major epitope on MLBs recognized by this antibody. Unfortunately, no band was detected in the MLB sample (data not shown). Many reasons could explain this result, especially given that the epitope recognized by the H36 antibody is a post-translational modification. It is likely that the epitope on the CDD protein was altered during the solubilization step used to extract the proteins from the MLBs.

### Comparison of the anti-SctA and H36 antibodies as MLB markers

**Fig 5:**
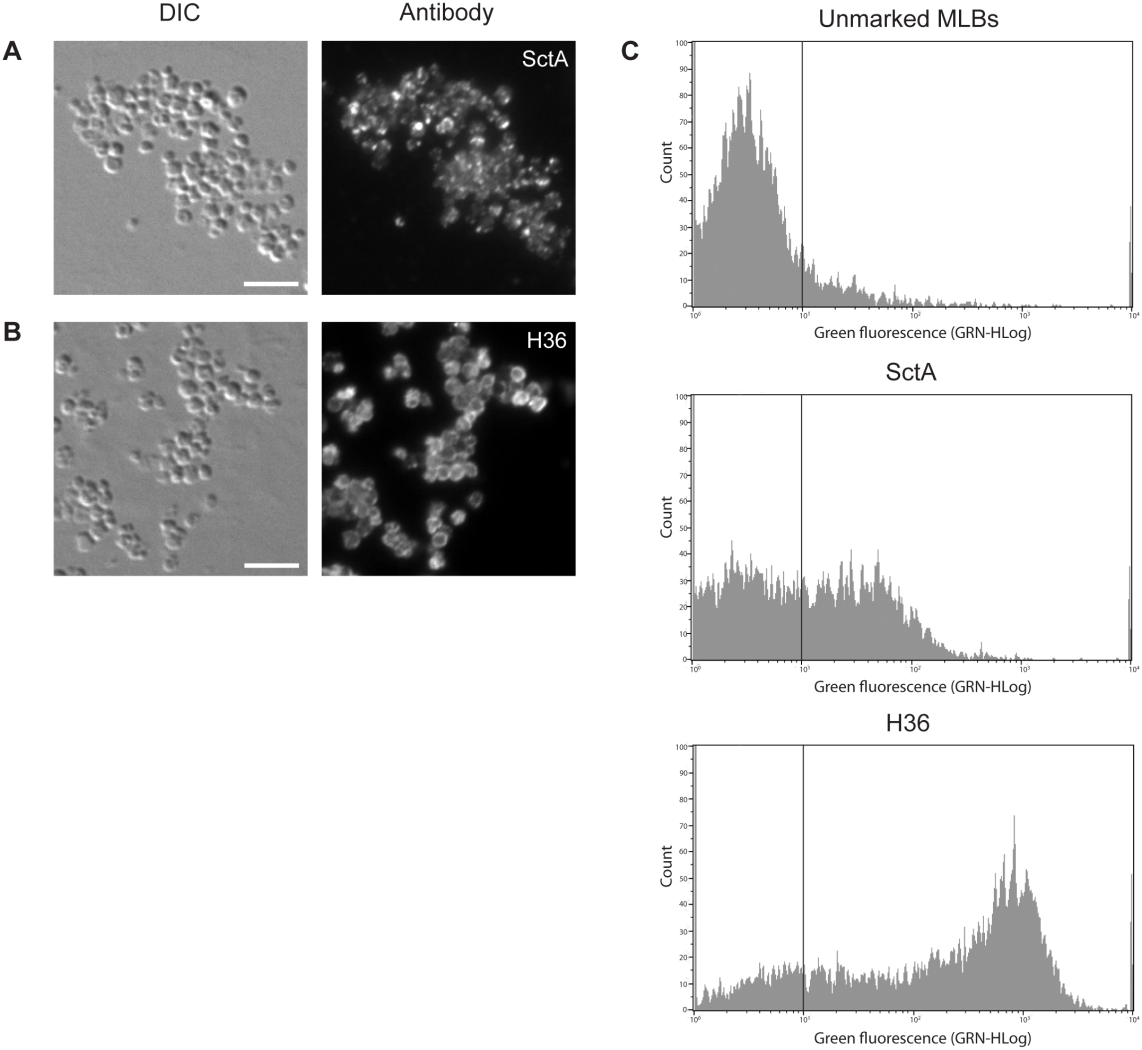
Comparison of the anti-SctA and H36 antibodies as MLB markers. Immunofluorescence analyses of purified MLBs using the (**A**) anti-SctA and (**B**) H36 antibodies. SctA labeling was not uniform and the protein appeared to be concentrated in aggregates in the MLBs. H36 labeling was only seen on the surface of MLBs, creating a ring-like appearance. The purified MLBs were deposited on glass slides and were processed for immunofluorescence using the anti-SctA or H36 antibodies. (**C**) Flow cytometric analysis of purified MLBs labeled with the anti-SctA or H36 antibody. Approximately 40% of the MLBs were labeled with the anti-SctA antibody while 60–70% were labeled with the H36 antibody. The fluorescence of the labeled MLBs was much more intense with the H36 antibody than with the anti-SctA antibody. Scale bar: A and B = 5 μm.

The H36 antibody may be a more suitable MLB marker than the anti-SctA antibody. The efficiency of the two antibodies as MLB markers was compared by immunofluorescence and flow cytometry. Fig 5 shows fluorescent microscopic images of purified MLBs labeled with the anti-SctA (A) and H36 (B) antibodies. In the case of the anti-SctA antibody, the labeling was not uniform and appeared as dots scattered throughout the MLBs. In the case of the H36 antibody, the labeling remained at the surface of the MLBs, giving them a ring-like appearance. The fluorescent signal was less intense for the anti-SctA antibody than for the H36 antibody.

The labeling efficiency and fluorescence intensity of the two MLB markers was also assessed in flow cytometric experiments (Fig 5C). Unmarked MLBs were used as a control to determine the gate delimiting unmarked and marked MLB. In the case of the anti-SctA antibody, only ~40% of the MLBs exhibited more intense fluorescence than the unlabeled MLB control. In the case of the H36 antibody, 60% to 70% of the MLBs exhibited more intense fluorescence than the unstained MLB control. In general, the fluorescence of the H36-labeled MLBs was more intense than that of the anti-SctA-labeled MLBs. This may because there are more H36 antibody binding sites on the MLBs or because they are more accessible than the binding sites of the anti-SctA antibody. The avidity of the H36 antibody for its epitope may also be higher than that of the anti-SctA antibody. These results confirmed the immunofluorescence results in that the labeling with the H36 antibody was more intense and more evenly distributed than that of the anti-SctA antibody.

## Discussion

The aim of the present study was to investigate the protein composition of MLBs in order to better understand the molecular mechanisms governing MLB formation in *D*. *discoideum*. Four major proteins associated with MLBs were identified (PonC, PhoPQ, SctA, and CDD). The presence of SctA was confirmed by Western blot and immunofluorescence analyses using an anti-SctA antibody. The protein was concentrated in aggregates in some MLBs while others appeared to contain less SctA. The CP7 protein was also identified as the main epitope of the H36 antibody, which was used as an MLB marker in a previous study [3]. However, CP7 was not detected on the MLBs. The H36 antibody likely binds to N-acetylglucosamine-1-phosphate given the high degree of similarity of the protein profiles detected by it and the AD7.5 antibody, which binds to N-acetylglucosamine-1-phosphate [18]. Other proteins were identified by the H36 antibody, including the CDD protein, which may be the epitope recognized on MLBs by this antibody. We showed that the H36 antibody was a better MLB marker that the anti-SctA antibody based on immunofluorescence and flow cytometric analyses.

To ensure that mass spectrometry is a reliable way to identify the MLB proteins, the analyses were performed three times and only proteins that were identified more than once were retained. This kind of filtering is necessary since multiple identifications by repeated analyses increases the confidence in the protein identifications and results in fewer false positives [32]. Proteins with a low peptide count were eliminated in order to reduce the number of false positives, a practice supported by experimental data [32]. Several protein categories that are more likely to give rise to false positives were also eliminated because they are commonly detected in proteomic studies. These categories include heat shock proteins, ribonucleoproteins, actin, ATP synthase, peroxiredoxins, tubulin, elongation factors, and phosphoglycerate mutase [23, 24]. Since many of the listed proteins are part of the minimal cellular stress proteome, it has been proposed that proteins that are detected by mass spectrometric analyses and that overlap this list should only be used as biomarkers for cellular stress [23].

As mentioned above, the aim of the present study was to better understand the formation of MLBs and their physiological role in *D*. *discoideum* by identifying proteins associated with MLBs. However, many of the proteins we detected have unknown functions or have not been well characterized. For example, the function of SctA is unknown, but was described in a study by Sabra et al. as a constitutively secreted protein associated with membranous materials (pycnosomes) [17]. They reported that SctA is mostly found in the extracellular medium rather than inside the cells. This finding and the fact that we were unsuccessful in our attempt to overexpress SctA in cells (data not shown) suggested that high intracellular concentrations of the protein may be toxic and that the cells may use pycnosomes or MLBs to secrete excess SctA. As observed in the present study and as Sabra et al. showed, SctA is present in dot-like structures within endosomal compartments, especially in post-lysosomes, in axenically grown cells. Given that intra-endosomal budding associated with MLB formation occurs in lysosomes and post-lysosomes, it is reasonable to conclude that some SctA-rich structures may be present in MLBs. This may explain why it is difficult, in immunofluorescence analyses, to distinguish between constitutively secreted SctA-rich pycnosomes and the smaller SctA-containing MLBs formed in the presence of digestible bacteria. In addition, the intra-endosomal pycnosomes described by Sabra et al. have a non-concentric multilamellar morphology similar to that of some MLBs containing regions of tangled lamellae (Fig 3). It is thus possible that the SctA proteins detected on MLBs may be associated with non-concentric membranes that may be remnants of pycnosomes. However, it is difficult to conclude that MLBs are a way of disposing of undesirable proteins since SctA can be secreted in the absence of MLBs.

As with SctA, the presence of the CDD protein on MLBs does not provide a clear indication about the role of these structures. The CDD protein contains a cytidine and deoxycytidylate deaminase region and a carbohydrate-binding domain according to a gene ontology annotation, but no further information is available in the literature or the databases. Since the CDD protein does not possess a transmembrane domain, it may be associated with MLBs through interactions with other glycosylated MLB proteins.

Ponticulin-like proteins (PonC) are similar to the ponticulin coded by the *ponA* gene, which is a membrane actin-binding protein with a nucleation capacity [33, 34]. It is tempting to assert that the PonC proteins detected on MLBs may be involved in actin-dependent invagination of lysosomes leading to the formation of MLBs. On the other hand, these PonC proteins may be false positives given their association with actin. As such, the presence of PonC proteins on MLBs should be verified using molecular tools such as specific antibodies. However, these tools are not currently available.

The presence of the PhoPQ protein on MLBs could, however, be of some significance. PhoPQ is a hypothetical protein similar to AprA, which is an autocrine repressor of proliferation secreted by growing cells [26]. The AprA protein functions as a chemorepellant for vegetative cells but not for starved cells [35]. By homology to the role of AprA, PhoPQ may provide a recognition signal of MLBs. For example, we do not know whether MLBs are reinternalized after secretion or whether there is a molecular signal that induces or prevents the phagocytosis of extracellular MLBs. We also do not know whether MLBs are intercellular communication “devices” or simply waste disposal structures. PhoPQ may in fact provide a molecular signal to the cells telling them what to do with extracellular MLBs. Future studies on reinternalization may provide clues about the physiological role of MLBs.

As mentioned in the introduction, a cysteine proteinase (ddCP38B) may be associated with secreted MLBs. Emslie *et al*. (1998) reported that MUD 62 and MUD 166 antibodies, which recognize type 3 *O*-linked glycosylations on many proteins, including ddCP38B, immunolabel intracellular and secreted MLBs [14]. Since ddCP38B was the main protein detected by the two antibodies, the authors concluded that ddCP38B is associated with MLBs. However, their results are very similar to ours with respect to the H36 antibody, which recognizes CP7, a protein that is not found on MLBs. It is possible that ddCP38B is not associated with MLBs and that another protein with a type 3 *O*-linked glycosylation on MLBs is recognized by the MUD 62 and MUD 166 antibodies. This other protein may in fact be one of the major proteins we detected in our analysis. We investigated this possibility using the DictyOGlyc 1.1 server [36]. It appeared that only the CDD protein has potential *O*-linked glycosylation sites. Based on this information and on the molecular weight of the CDD protein, the protein reported by Emslie et al. may in fact have been the CDD protein. Lastly, we did not detect a cysteine proteinase based on our results from the mass spectrometric analysis of purified MLBs.

Discoidin I, an endogenous lectin involved in cell-substratum adhesion and ordered cell migration [37], has also been detected on MLBs in a previous study [12]. This protein is synthesized and secreted soon after the cells are starved and binds to glycoconjugates containing N-acetylgalactosamine or galactose [38]. It was suggested that the MLBs may in fact serve as vehicles for transporting discoidin I to the extracellular space where it exerts its function [12] in a process similar to that of lung surfactant externalized in lamellar bodies by type II cells [39]. We only detected discoidin I once in our three mass spectrometric analyses (data not shown). However, since it is only present in cells entering the multicellular stage, it implies that MLBs formed at the vegetative state will not contain it. The presence of discoidin I on MLBs also depends on the ingestion of bacterial glycoconjugate ligands by the cells. Indeed, MLBs produced by amoebae fed boiled *Klebsiella pneumoniae* cells (which retain their discoidin I ligand) contained the lectin, while MLBs produced by amoebae fed boiled *Escherichia coli* cells (which had lost their discoidin I ligand) did not [40].

The present study was limited by several aspects of secreted and transmembrane proteins. These proteins are subject to glycosylation, which can affect peptide detection by mass spectrometry. It is thus likely that some proteins associated with MLBs were not detected due to post-translational modifications. This is especially true for proteins with low molecular weights whose identification is more susceptible to be affected by post-translational modifications, as it may be the case for the 10 kDa band of the MLB protein profile (Fig 1). Moreover, MLBs are highly resistant lipidic structures from which it is difficult to extract and solubilize transmembrane proteins. The denaturation and gel migration solutions may also have interfered with the detection of peptides and the recognition of epitopes by the antibodies in the Western blot analyses. Furthermore, the compact structures of MLBs may have reduced the accessibility of epitopes in the immunofluorescence analyses. Lastly, fusing GFP genes or other tags with secreted or transmembrane protein genes may alter protein functions or locations. In the present study, we produced a GFP-fused version of SctA but were unable to detect accumulations of these proteins in cells or in MLBs, possibly due to the toxicity of the overexpressed protein (data not shown). For all these reasons, we do not consider that our list of MLB-associated proteins is complete and other proteins may be identified in the future using other experimental strategies.

The present study provided new biochemical clues about the composition of MLBs. The functional characterization of the four major MLB proteins that we identified is of great interest. Elucidating the molecular mechanism governing MLB formation is essential to better understand the bacteria packaging process and, as such, the transmission and persistence of pathogenic bacteria. Future studies should also investigate whether the biochemical composition of MLBs varies with the food source given that the type of ingested bacteria can affect the morphology of the MLBs [7].

## Supporting Information

S1 Table Complete list of proteins associated with MLBs based on mass spectrometric analyses. Proteins identified only once and with three peptide counts or fewer are not listed in the table.
(PDF)

S2 Table Proteins identified by mass spectrometry after immunoprecipitation with H36. (XLS)

## References

[1] Schmitz G, Muller G. Structure and function of lamellar bodies, lipid-protein complexes involved in storage and secretion of cellular lipids. J Lipid Res. 1991;32(10):1539–70. . 1797938

[2] Mercer EH, Shaffer BM. Electron Microscopy of Solitary and Aggregated Slime Mould Cells. The Journal of biophysical and biochemical cytology. 1960;7(2):353–6. 19866567 10.1083/jcb.7.2.353 PMC2224802

[3] Paquet VE, Lessire R, Domergue F, Fouillen L, Filion G, Sedighi A, et al Lipid composition of multilamellar bodies secreted by *Dictyostelium discoideum* reveals their amoebal origin. Eukaryot Cell. 2013;12(10):1326–34. 10.1128/EC.00107-13 23748431 PMC3811330

[4] Gezelius K. Further studies in the ultrastructure of Acrasiae. Experimental cell research. 1961;23(2):300–10. 10.1016/0014-4827(61)90039-8

[5] Hohl HR. Nature and Development of Membrane Systems in Food Vacuoles of Cellular Slime Molds Predatory upon Bacteria. J Bacteriol. 1965;90(3):755–65. 16562078 10.1128/jb.90.3.755-765.1965 PMC315722

[6] Marchetti A, Mercanti V, Cornillon S, Alibaud L, Charette SJ, Cosson P. Formation of multivesicular endosomes in *Dictyostelium*. J Cell Sci. 2004;117(Pt 25):6053–9. 10.1242/jcs.01524 . 15536120

[7] Denoncourt AM, Paquet VE, Charette SJ. Potential role of bacteria packaging by protozoa in the persistence and transmission of pathogenic bacteria. Frontiers in microbiology. 2014;5:240 10.3389/fmicb.2014.00240 24904553 PMC4033053

[8] Berk SG, Ting RS, Turner GW, Ashburn RJ. Production of respirable vesicles containing live *Legionella pneumophila* cells by two *Acanthamoeba* spp. Appl Environ Microbiol. 1998;64(1):279–86. Epub 1998/01/22. 9435080 10.1128/aem.64.1.279-286.1998 PMC124706

[9] Koubar M, Rodier MH, Garduno RA, Frere J. Passage through *Tetrahymena tropicalis* enhances the resistance to stress and the infectivity of *Legionella pneumophila*. FEMS Microbiol Lett. 2011;325(1):10–5. Epub 2011/11/19. 10.1111/j.1574-6968.2011.02402.x . 22092856

[10] Raghu Nadhanan R, Thomas CJ. *Colpoda* secrete viable *Listeria monocytogenes* within faecal pellets. Environ Microbiol. 2014;16(2):396–404. Epub 2013/08/29. 10.1111/1462-2920.12230 . 23981071

[11] Brandl MT, Rosenthal BM, Haxo AF, Berk SG. Enhanced survival of *Salmonella enterica* in vesicles released by a soilborne *Tetrahymena* species. Appl Environ Microbiol. 2005;71(3):1562–9. Epub 2005/03/05. 71/3/1562 [pii] 10.1128/AEM.71.3.1562-1569.2005 15746361 PMC1065168

[12] Barondes SH, Haywood-Reid PL, Cooper DN. Discoidin I, an endogenous lectin, is externalized from *Dictyostelium discoideum* in multilamellar bodies. J Cell Biol. 1985;100(6):1825–33. 2581974 10.1083/jcb.100.6.1825 PMC2113611

[13] Fukuzawa M, Ochiai H. Different subcellular localizations of discoidin I monomer and tetramer in *Dictyostelium discoideum* cells: using conformation-specific monoclonal antibodies. Experimental cell research. 1993;204(1):61–72. 10.1006/excr.1993.1009 . 8416797

[14] Emslie KR, Birch D, Champion AC, Williams KL. Localisation of glycoproteins containing type 3 O-linked glycosylation to multilamellar bodies in *Dictyostelium discoideum*. Eur J Protistol. 1998;34(3):321–8. ISI:000076499200012.

[15] Mercanti V, Charette SJ, Bennett N, Ryckewaert JJ, Letourneur F, Cosson P. Selective membrane exclusion in phagocytic and macropinocytic cups. J Cell Sci. 2006;119(Pt 19):4079–87. Epub 2006/09/14. jcs.03190 [pii] 10.1242/jcs.03190 . 16968738

[16] Cornillon S, Pech E, Benghezal M, Ravanel K, Gaynor E, Letourneur F, et al Phg1p is a nine-transmembrane protein superfamily member involved in *Dictyostelium* adhesion and phagocytosis. J Biol Chem. 2000;275(44):34287–92. 10.1074/jbc.M006725200 . 10944536

[17] Sabra A, Leiba J, Mas L, Louwagie M, Couté Y, Journet A, et al Pycnosomes: condensed endosomal structures secreted by *Dictyostelium* amoebae. Submitted to PLOS ONE Manuscript number: PONE-D-16-05334. 2016. 10.1371/journal.pone.0154875 PMC4871501 27187592

[18] Mehta DP, Ichikawa M, Salimath PV, Etchison JR, Haak R, Manzi A, et al A lysosomal cysteine proteinase from *Dictyostelium discoideum* contains N-acetylglucosamine-1-phosphate bound to serine but not mannose-6-phosphate on N-linked oligosaccharides. J Biol Chem. 1996;271(18):10897–903. . 8631906 10.1074/jbc.271.18.10897

[19] Smith EW, Lima WC, Charette SJ, Cosson P. Effect of starvation on the endocytic pathway in *Dictyostelium* cells. Eukaryot Cell. 2010;9(3):387–92. 10.1128/EC.00285-09 20097741 PMC2837978

[20] Rabilloud T. Solubilization of proteins in 2-D electrophoresis. An outline. Methods in molecular biology. 1999;112:9–19. . 10027223 10.1385/1-59259-584-7:9

[21] Molloy MP, Herbert BR, Walsh BJ, Tyler MI, Traini M, Sanchez JC, et al Extraction of membrane proteins by differential solubilization for separation using two-dimensional gel electrophoresis. Electrophoresis. 1998;19(5):837–44. 10.1002/elps.1150190539 . 9629924

[22] Rabilloud T. Use of thiourea to increase the solubility of membrane proteins in two-dimensional electrophoresis. Electrophoresis. 1998;19(5):758–60. 10.1002/elps.1150190526 . 9629911

[23] Wang P, Bouwman FG, Mariman EC. Generally detected proteins in comparative proteomics—a matter of cellular stress response? Proteomics. 2009;9(11):2955–66. 10.1002/pmic.200800826 . 19415655

[24] Petrak J, Ivanek R, Toman O, Cmejla R, Cmejlova J, Vyoral D, et al Deja vu in proteomics. A hit parade of repeatedly identified differentially expressed proteins. Proteomics. 2008;8(9):1744–9. 10.1002/pmic.200700919 . 18442176

[25] Wuestehube LJ, Luna EJ. F-actin binds to the cytoplasmic surface of ponticulin, a 17-kD integral glycoprotein from *Dictyostelium discoideum* plasma membranes. J Cell Biol. 1987;105(4):1741–51. 3312238 10.1083/jcb.105.4.1741 PMC2114643

[26] Brock DA, Gomer RH. A secreted factor represses cell proliferation in *Dictyostelium*. Development. 2005;132(20):4553–62. 10.1242/dev.02032 16176950 PMC4484793

[27] Charette SJ, Cosson P. Altered composition and secretion of lysosome-derived compartments in *Dictyostelium* AP-3 mutant cells. Traffic. 2008;9(4):588–96. 10.1111/j.1600-0854.2008.00706.x . 18194410

[28] Rauchenberger R, Hacker U, Murphy J, Niewohner J, Maniak M. Coronin and vacuolin identify consecutive stages of a late, actin-coated endocytic compartment in *Dictyostelium*. Curr Biol. 1997;7(3):215–8. . 9276759 10.1016/s0960-9822(97)70093-9

[29] Ord T, Adessi C, Wang L, Freeze HH. The cysteine proteinase gene cprG in *Dictyostelium discoideum* has a serine-rich domain that contains GlcNAc-1-P. Archives of biochemistry and biophysics. 1997;339(1):64–72. 10.1006/abbi.1996.9870 . 9056234

[30] Gustafson GL, Thon LA. Purification and characterization of a proteinase from *Dictyostelium discoideum*. J Biol Chem. 1979;254(24):12471–8. . 500725

[31] Mehta DP, Etchison JR, Freeze HH. Characterization, subcellular localization, and developmental regulation of a cysteine proteinase from *Dictyostelium discoideum*. Archives of biochemistry and biophysics. 1995;321(1):191–8. 10.1006/abbi.1995.1385 . 7639520

[32] Elias JE, Haas W, Faherty BK, Gygi SP. Comparative evaluation of mass spectrometry platforms used in large-scale proteomics investigations. Nature methods. 2005;2(9):667–75. 10.1038/nmeth785 . 16118637

[33] Hitt AL, Lu TH, Luna EJ. Ponticulin is an atypical membrane protein. J Cell Biol. 1994;126(6):1421–31. 8089175 10.1083/jcb.126.6.1421 PMC2290967

[34] Hitt AL, Hartwig JH, Luna EJ. Ponticulin is the major high affinity link between the plasma membrane and the cortical actin network in *Dictyostelium*. J Cell Biol. 1994;126(6):1433–44. 8089176 10.1083/jcb.126.6.1433 PMC2290950

[35] Phillips JE, Gomer RH. A secreted protein is an endogenous chemorepellant in *Dictyostelium discoideum*. Proc Natl Acad Sci U S A. 2012;109(27):10990–5. 10.1073/pnas.1206350109 22711818 PMC3390837

[36] Gupta R, Jung E, Gooley AA, Williams KL, Brunak S, Hansen J. Scanning the available *Dictyostelium discoideum* proteome for O-linked GlcNAc glycosylation sites using neural networks. Glycobiology. 1999;9(10):1009–22. . 10521537 10.1093/glycob/9.10.1009

[37] Springer WR, Cooper DN, Barondes SH. Discoidin I is implicated in cell-substratum attachment and ordered cell migration of *Dictyostelium discoideum* and resembles fibronectin. Cell. 1984;39(3 Pt 2):557–64. . 6509552 10.1016/0092-8674(84)90462-8

[38] Rosen SD, Kafka JA, Simpson DL, Barondes SH. Developmentally regulated, carbohydrate-binding protein in *Dictyostelium discoideum*. Proc Natl Acad Sci U S A. 1973;70(9):2554–7. 4517669 10.1073/pnas.70.9.2554 PMC427054

[39] Goerke J. Lung surfactant. Biochim Biophys Acta. 1974;344(3–4):241–61. . 4613380 10.1016/0304-4157(74)90009-4

[40] Cooper DNW, Haywood-Reid PL, Springer WR, Barondes SH. Bacterial glycoconjugates are natural ligands for the carbohydrate binding site of discoidin I and influence its cellular compartmentalization. Developmental biology. 1986;114(2):416–25. 10.1016/0012-1606(86)90206-X

