# Supplementary material for: Identification of Proteins Associated with Multilamellar Bodies Produced by Dictyostelium discoideum
Source: PLoS One. 2016 Jun 24;11(6):e0158270. doi: 10.1371/journal.pone.0158270 (PMC4920372; doi:10.1371/journal.pone.0158270)
Supplement: S1 Table — Proteins identified only once and with three peptide counts or fewer are not listed in the table. (PDF) [file pone.0158270.s001.pdf]

S1 Table. Complete list of proteins associated with MLBs based on mass spectrometric analyses. Proteins identified only once and with three peptide counts or fewer are not listed in the table.

Protein	Description	Gene ID	Molecular weight (kDa)	Number of identifications on 3 analyses
**PonC**	Ponticulin-like protein	DDB_G0286717<br>DDB_G0286715<br>DDB_G0286721<br>DDB_G0286719<br>DDB_G0286723	15	3
**PhoPQ**	PhoPQ-activated pathogenicity-related protein	DDB_G0276897	56	3
**SctA**	Secreted Protein A	DDB_G0278725	18	3
**EfbA**	Elongation factor 2	DDB_G0288373	93	3
**rpl13**	S60 ribosomal protein L13	DDB_G0291870	24	3
**rpl13a**	S60 ribosomal protein L13a	DDB_G0275881	21	3
**CDD**	Cytidine/deoxycytidylate deaminase zinc-binding domain-containing protein	DDB_G0292096	35	3
**rpl4**	60S ribosomal protein L4	DDB_G0277803	40	3
**act1**	Major actin	DDB_G0289553	42	2
**Ef1A**	Elongation factor 1 alpha	DDB_G0269134<br>DDB_G0269136	50	2
**ancA**	ADP/ATP translocase	DDB_G0267454	33	2
**mvpB**	Major vault protein B	DDB_G0291127	95	2
**mppB**	Mitochondrial processing peptidase beta subunit	DDB_G0288777	53	2
**ndkC**	Nucleoside diphosphate kinase	DDB_G0273069<br>DDB_G0273805	17	2
**rps4**	40S ribosomal protein S4	DDB_G0277975	17	2

**vatM**	V-ATPase subunit M	DDB_G0291858	93	2
**rpl14**	S60 ribosomal protein L14	DDB_G0277975	17	2
**atp1**	ATP synthase	DDB_G0294012	57	2
**Prdx4**	Peroxiredoxin 4	DDB_G0274859	29	2
**mvpA**	Major vault protein-alpha	DDB_G0269156	94	2
**pGAM**	Phosphoglycerate mutase	DDB_G0285311	28	2
**rpl36**	S60 ribosomal protein L36	DDB_G0271668	12	2
**rpl28**	60S ribosomal protein L28	DDB_G0282379	14	2
**rps8**	40S ribosomal protein S8	DDB_G0291864	24	2
**efa1G**	Elongation Factor 1 Gamma	DDB_G0282979	47	2
**rpl21**	S60 ribosomal protein L21	DDB_G0279387	18	2
**tubB**	Beta tubulin	DDB_G0269196	51	2
**rps9**	40S ribosomal protein S9	DDB_G0289877	21	2
**Carboxylesterase**	Carboxylesterase, type B family protein	DDB_G0279717	59	2
**H2AX**	Histone H2A	DDB_G0279667	17	2
**act3**	Actin	DDB_G0289487	42	1
**rps18**	40S ribosomal protein S18	DDB_G0276415	18	1
**porA**	Porin	DDB_G0271848	30	1
**atp5b**	ATP synthase Beta subunit	DDB_G0269916	71	1
**Hsp70**	Heat shock protein 70	DDB_G0273249 DDB_G0273623	70	1
**mshp70**	Stress-70 protein, mitochondrial	DDB_G0293298	71	1
**aatA**	Aspartate aminotransferase	DDB_G0268664	47	1
